# Broken Spinal Needle in a Morbidly Obese Parturient Presenting for Urgent Cesarean Section

**DOI:** 10.1155/2020/8880464

**Published:** 2020-09-30

**Authors:** Saurin J. Shah, Kristen Vanderhoef, Michael Ibrahim

**Affiliations:** Department of Anesthesiology, University of Florida College of Medicine Jacksonville, Jacksonville, FL, USA

## Abstract

Neuraxial anesthesia has become the preferred method of anesthesia for nonemergent cesarean delivery and cases where regional anesthesia is not contraindicated. Multiple cases of broken spinal and epidural needles have been reported in the literature over the last several years; however, the specific incidence of needle breakage is still unknown. Less reliance on general anesthesia and increasing parturient body mass index (BMI) has likely contributed to more reports of broken needles during regional anesthesia for obstetric surgery. We describe a case of a broken spinal needle after attempted spinal anesthetic placement for cesarean delivery in a morbidly obese parturient, subsequent postoperative management, and current treatment recommendations.

## 1. Introduction

Obesity is a known risk factor for cesarean sections (c/s). Increasing BMI has been strongly associated with a higher rate of sections [1]. Morbidly obese parturients pose many challenges to the anesthesiologist; spinal anesthesia is the preferred anesthetic of choice for c/s to minimize maternal fetal risks associated with general anesthesia [2]. Placement of spinal anesthesia may be difficult in obese patients.

Spinal anesthesia is associated with several complications that may occur with different incidences. Technique, body habitus, co-morbidities, patient selection are a few of the many co-factors that may contribute to the multifactorial nature of risks and complications associated with neuraxial anesthesia. Complications may be as minor as nausea/vomiting and shivering, moderate such as postdural puncture headaches (PDPH), or major such as direct needle trauma, spinal cord injury, or infections [3]. The incidence of most of the major complications that may occur after spinal anesthesia is very rare. Complications such as nausea and vomiting have reported incidences as high as 80% in obstetric patients, while PDPHs occur about 1-2% of the time after the administration of spinal anesthesia or inadvertent dural puncture after epidural procedure [3]. The incidence of broken spinal needles is not well reported [4]; however, more cases of broken needles in obstetric patients have been noted in the literature over the last decade.

## 2. Case Report

A 37-year-old gravida 2, para 1 (previous c/s), 37-week-pregnant woman with a BMI of 61.1 kg/m^2^ was admitted for pre-eclampsia with elevated blood pressures. The patient was scheduled for urgent c/s upon admission and evaluation by the obstetric service, due to inability to control patient's blood pressure and her history of one prior cesarean delivery. Significant findings on history and physical revealed a female in no apparent distress, elevated blood pressures (160's/100's), BMI as noted previously, Mallampati class 3, no evidence of fetal distress, and normal laboratory values. After a thorough preoperative evaluation, informed consent was obtained for epidural anesthesia.

The patient was transported to the procedure room, moved themselves over to operating table, and assisted into the sitting position for epidural placement. ASA standard monitors were placed with normal vital signs; fetal heart tones were within normal limits. A traditional medial approach was used to place an epidural catheter at the L1-L2 interspace; using a 17-gauge 5-inch Tuohy needle, the epidural catheter was placed without difficulty with a loss of resistance at 9 cm. Local anesthetic was administered in divided doses with resultant one-sided block; appropriate maneuvers were unsuccessful in obtaining a bilateral block adequate for surgical anesthesia. Decision was made by the senior anesthesiologist o remove epidural catheter and proceed with a single-shot spinal anesthetic at the L1-L2 interspace. The patient was reprepped and draped. A 24-gauge 4.5-inch Whitacre needle was inserted via a metal introducer. Resistance was met, and the needle was withdrawn with difficulty; it was observed that approximately ½ of the needle was missing upon removal from the introducer. The introducer was removed, neurologic exam was intact, and vital signs were stable. After consultation with OB and neurosurgery, decision was made to proceed with c/s under general endotracheal anesthesia (GETA) due to continued elevated blood pressures.

**Figure 1:**
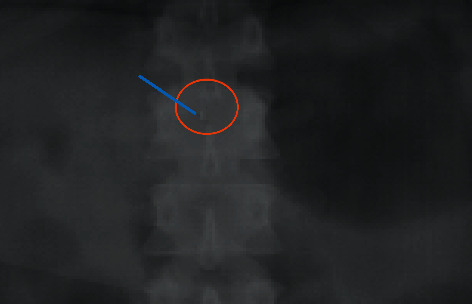
AP lumbar spine radiograph.

**Figure 2:**
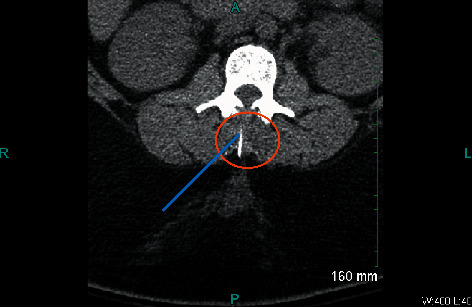
Computed tomography of lumbar spine.

After the procedure, plain radiographs were obtained to locate the needle fragment (Figure 1). Obtaining and reviewing images were difficult due to patient's large body habitus. The patient was seen and evaluated by Neurosurgery the following day; a CT scan was performed showing “foreign body” posterolateral to the left lamina of L1 (Figure 2). Neurosurgery recommended no intervention due to lack of symptoms and location of needle behind bony structures. At patient's request, second consultation was performed by the orthopedic spine service; decision made to remove the needle in the operating room the following day. A 1-inch bent needle fragment was removed under fluoroscopy guidance without complications. The patient was discharged home on POD 1 (POD 2 c/s).

## 3. Discussion

The incidence of broken spinal needles is not known. However, certain risk factors may increase the chances of needle damage during placement. Emergency procedures, morbid obesity, and multiple puncture attempts all may increase the chances of a broken needle. Increased resistance during placement and redirection of the needle without appropriate mobilization of the introducer has been recognized in some of the case reports describing broken needles. Lonnée and Fasting reported 3 cases of broken spinal needles in obese patients (BMI > 30 kg/m^2^) after meeting bony resistance during multiple failed attempts [5]. Greenway et al. reported the occurrence of a microtip fracture during attempted placement of a 25 Ga spinal needle in a morbidly obese patient (BMI >50 kg/m^2^) [6]. Poor differentiation of landmarks, redirection of the needle without concomitant redirection of the introducer, and resistance to needle advancement were all considered as possible factors contributing to failed placement and needle breakage. Patient selection, potential use of larger gauge needles, withdrawing the spinal needle into the introducer prior to redirection, and utilization of ultrasound have all been proposed to reduce the possibility of a broken needle [5]. Preinsertion lumbar ultrasound has been shown to reduce the number of puncture attempts and increase the success rate of spinal placement in obese patients [1, 7]. Chin et al. reported a 2 to 1 first-time success rate when performing spinal anesthesia under ultrasound guidance vs. landmarks only in patients with BMI >35 kg/m^2^ [8]. Shaylor et al. reported even higher success rates for the first-time placement of spinal anesthesia in both obese and nonobese patients [9].

Past literature is split regarding the needle fragment removal. Normal neurologic exam and no direct migration path towards the spinal canal are used as determinants for the need for surgical intervention. More recently, current literature suggests that the patient is likely to remain symptom-free if the needle fragment is removed soon after the initial breakage.

Morbid obesity can make the placement of spinal anesthesia and perioperative management very challenging in the obstetrics population. Utilization of ultrasound as well as adherence to proper technique may significantly reduce the risk of complications during spinal placement in morbidly obese parturients.
